# Supplementary material for: Genetic dissection of MutL complexes in Arabidopsis meiosis
Source: Nucleic Acids Res. 2025 Mar 19;53(5):gkaf187. doi: 10.1093/nar/gkaf187 (PMC11920794; doi:10.1093/nar/gkaf187)
Supplement: gkaf187_Supplemental_Files [file gkaf187_supplemental_files.zip › Kbiri_Suppl_Figs_R1.pdf]

Supplementary figures

Genetic dissection of MutL complexes in Arabidopsis meiosis

Nadia Kbiri, Nadia Fernández-Jiménez, Wojciech Dziegielewski, Esperanza Sáez-Zárate, Alexandre Pelé, Ana Mata-Villanueva, Julia Dluzewska, Juan L. Santos, Mónica Pradillo and Piotr A. Ziolkowski

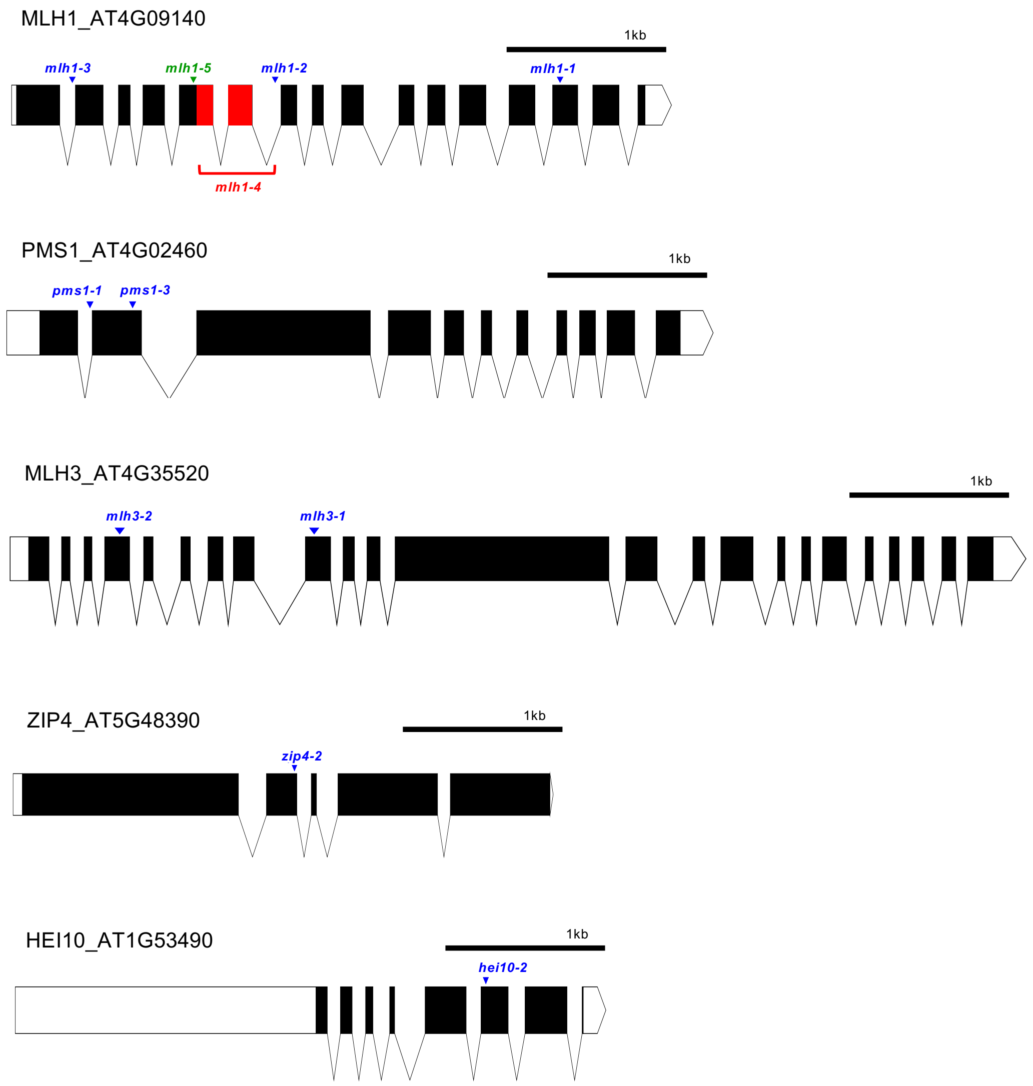

Supplementary Figure S1. Scaled representation of the genomic sequences of the MutL genes *MLH1, PMS1,* and *MLH3* and the ZMM genes *ZIP4* and *HEI10*. 3' and 5' UTRs are represented by white blocks, exons by black blocks, and introns by V-shaped linkers. T-DNA insertion mutations are represented by blue arrowheads and text, the SNP mutation is represented by a green arrowhead and text, and the deletion mutation is represented by a red bracket, text, and deleted exons. AGI codes are provided. Scale bar, 1kb.

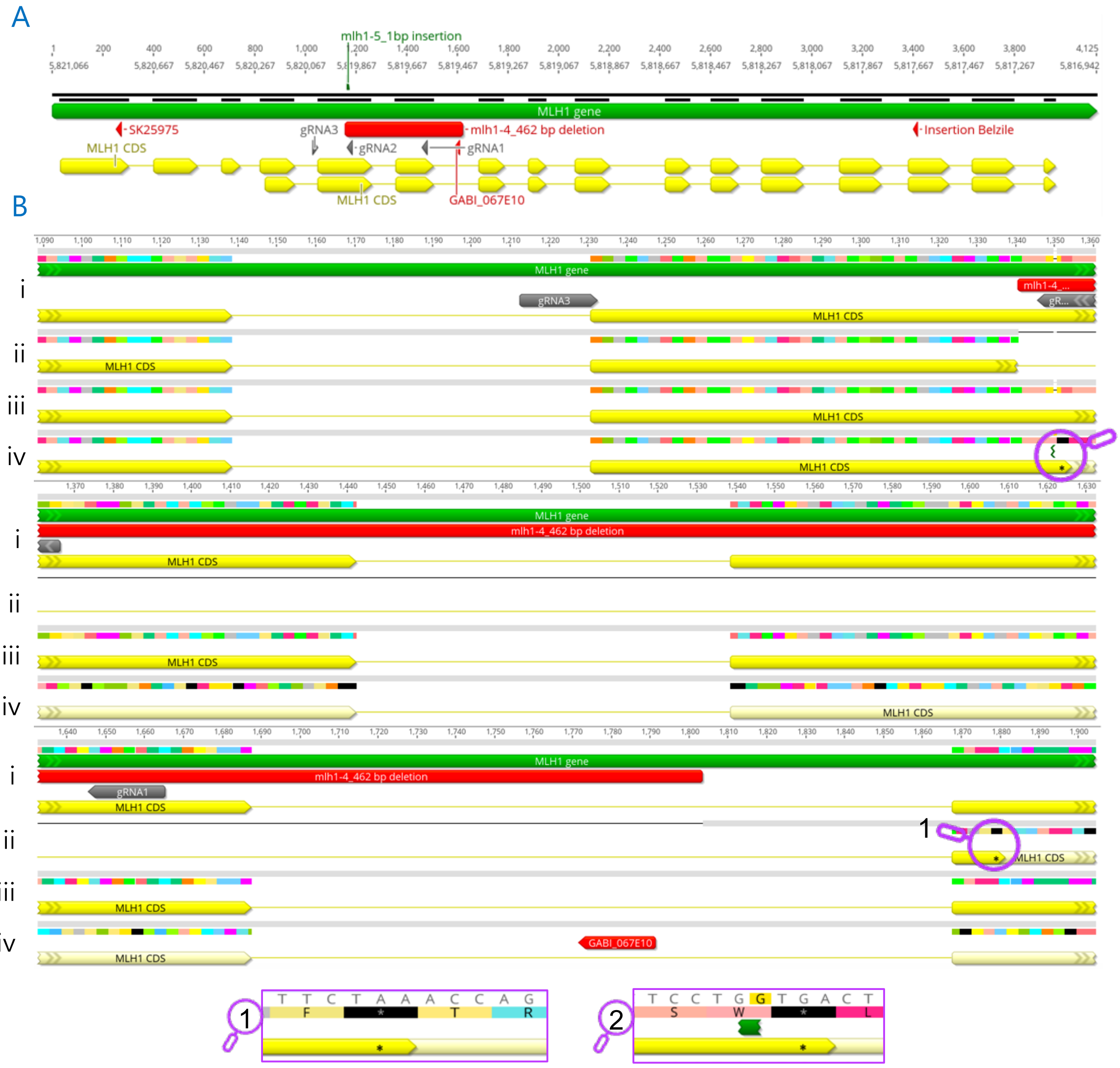

Supplementary Figure S2. ***mlh1-4* and *mlh1-5* CRISPR-Cas9 mediated mutants in Col-0 and L*er*-0 respectively. A**, *MLH1* gene structure. The exons are represented with yellow arrows, the gRNAs used for the mutagenesis in grey arrowheads, the obtained 462 bp deletion in Col-0 is represented with a red rectangle, and the 1 bp insertion in Ler-0 is represented with a green flag. The position of the T-DNA insertion of the other MLH1 mutants is also represented with red arrowheads. The genomic position is represented with the scale. **B**, *mlh1-4* and *mlh1-5* are aligned to the wildtype references of Col-0 and L*er*-0. **i.** Col-0 WT*.* **ii.** *mlh1-4* allele. **iii.** L*er*-0 WT. iv. *mlh1-5* allele*.* The 462 bp genomic and 250 bp in coding sequence deletion, in Col-0, introduces a frameshift and multiple STOP codons. Similarly, the 1bp insertion in L*er*-0 also introduces a frameshift and multiple STOP codons. STOP codons are represented in black, the predicted first STOP codons and the predicted end of the transcripts, are highlighted with purple magnifying glasses: **1** for *mlh1-4* and **2** for *mlh1-5*. The reverse sequence is used for simplicity.

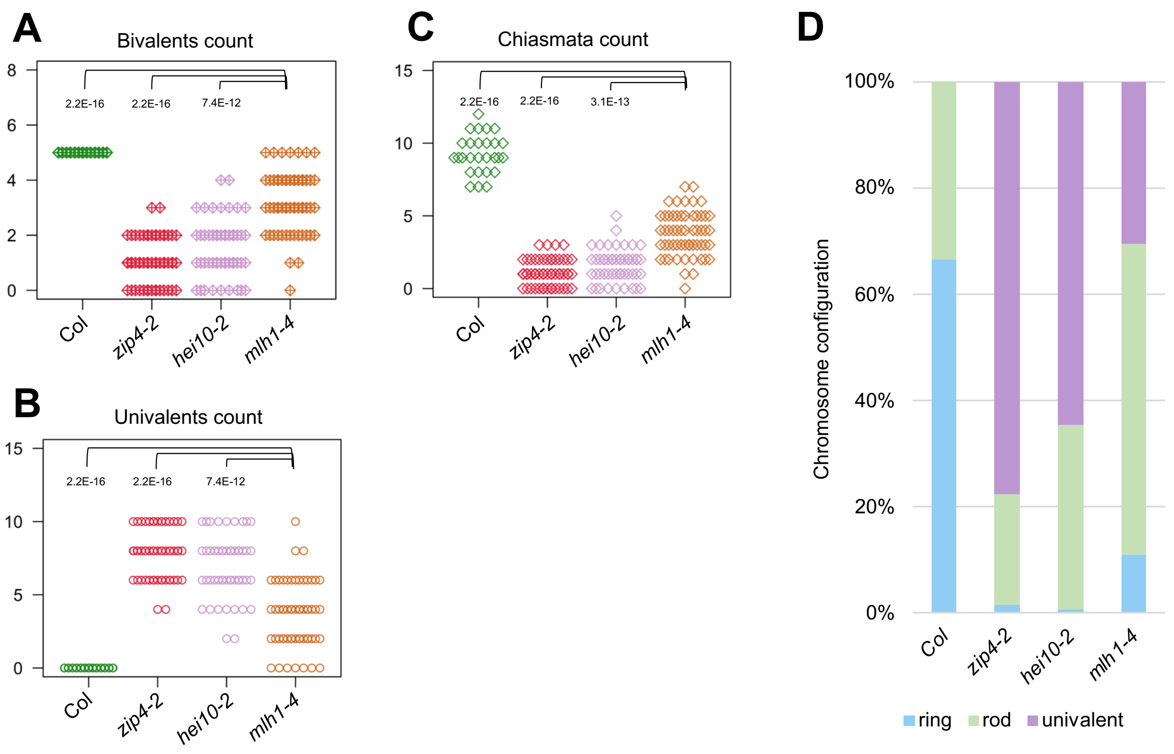

Supplementary Figure S3. Comparative analysis of the meiotic behavior of Col, *zip4-2, hei10-2,* and *mlh1-4.* A-C. Graphical representation of bivalent count per metaphase I meiocyte (**A**), univalent count (**B**), and chiasmata count (**C**). The *P* values were estimated using a Welch t-test. **D.** Chromosome configuration represented in ring, rod, and univalent proportions. A summary table of the average numbers of univalents, bivalents, and chiasmata is provided in Supplementary Table S6.

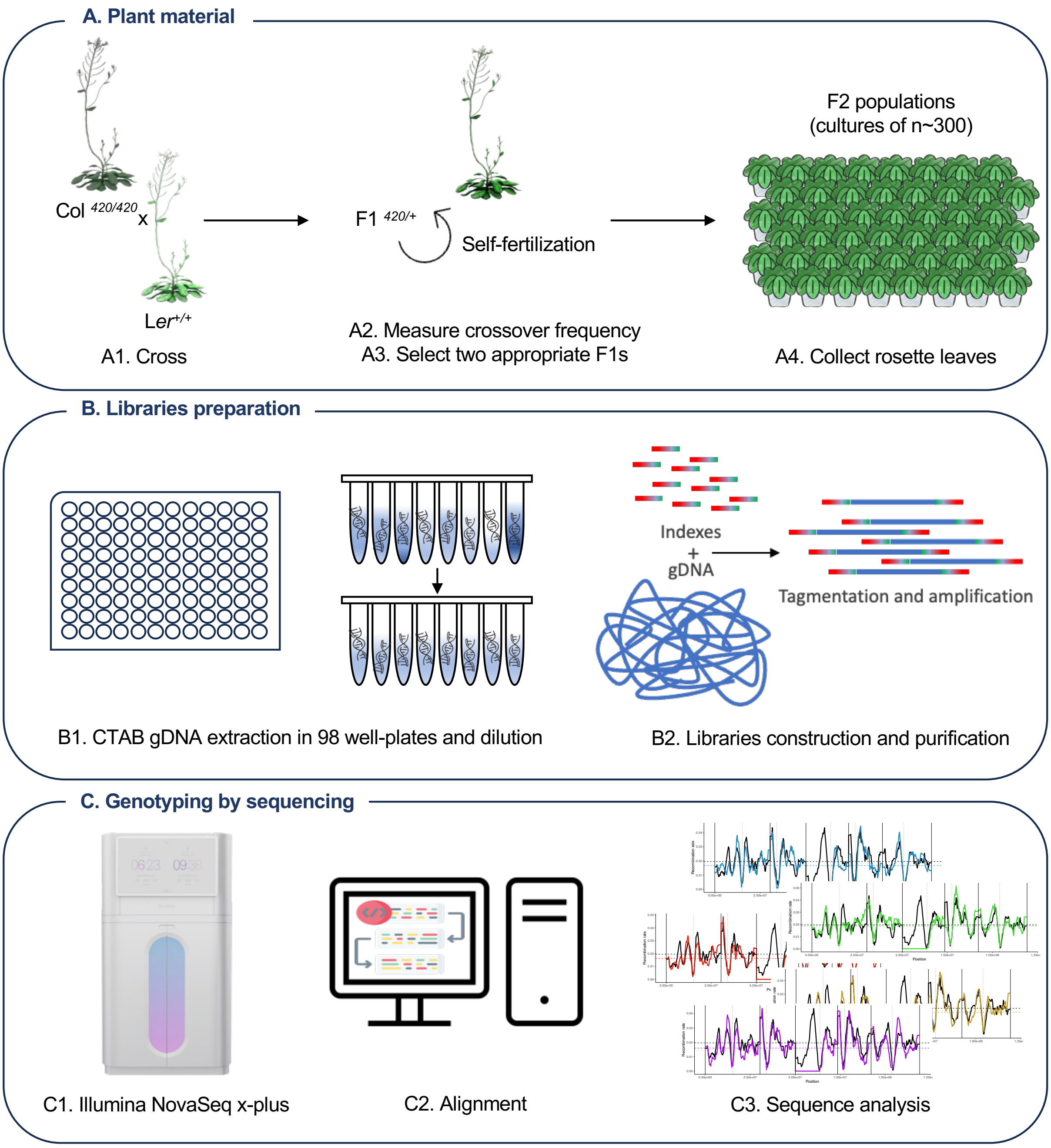

Supplementary Figure S4. Generation of genome-wide crossover maps. A. Plant material preparation, the Col$^{420/420}$ and *Ler*$^{+/+}$ Arabidopsis genetic background were chosen to generate the appropriate mutants and then crossed to create an $F_1^{420/+}$ population (**A1**). The $F_1^{420/+}$ were grown to seed, crossover recombination frequency was measured in the 420 interval (**A2**), and 2 individuals were selected based on having an RF closest to the average of the population and the best fluorescent tags segregation ratios (**A3**). Next, about 300 seeds were sown to construct an F_2 population and rosette leaves were collected for gDNA extraction. **B.** gDNA libraries were constructed by extracting gDNA from rosette leaves using CTAB protocol. The DNA samples were diluted to a concentration of 5ng/µL (**B1**), then tagmented using Tn5 and amplified with KAPA2G Robust to introduce indexes. The pooled, size selected (450bp-

700bp) and purified libraries were then sent for sequencing (**B2**). **C.** gDNA libraries were sequenced using NovaSeq x-plus and demultiplexed by Macrogen Europe (**C1**), the received data was aligned to a Col reference and computed (**C2**) to allow for different types of sequence analyses (**C3**). The detailed computation of genome-wide sequencing is provided in materials and methods. Rights to the Arabidopsis plant drawing belong to @_HETAKA, https://doi.org/10.7875/togopic.2021.057, the plant in a pot was designed by Freepik, and the Illumina NovaSeq X-10 was taken from Illumina's official website https://emea.illumina.com.

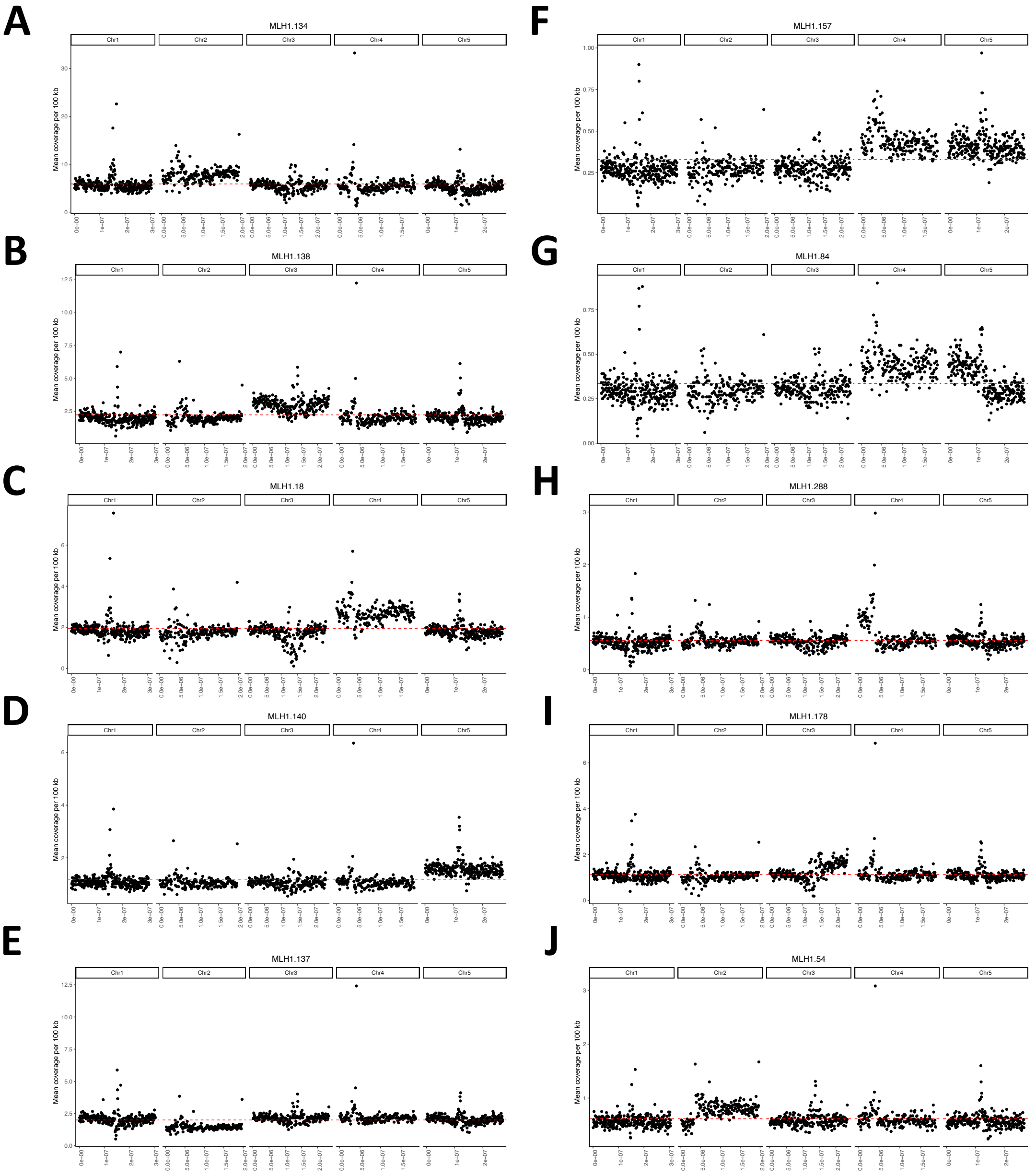

Supplementary Figure S5. Examples of all types of aneuploidy found in Col/L*er* *mlh1* F_2 individuals. Mean sequence coverage plots per 100 kb. **A.** Trisomy of chromosome 2. **B.** Trisomy of chromosome 3 . **C.** Trisomy of chromosome 4. **D.** Trisomy of chromosome 5. **E.** Monosomy of chromosome 2. **F.** Trisomy of chromosomes 4 and 5. **G.** Trisomy of chromosome 4 and north arm of chromosome 5. **H.** Partial trisomy of north arm of chromosome 4. **I.** Partial trisomy of south arm of chromosome 3. **J.** Partial trisomy of south arm of chromosome 2.

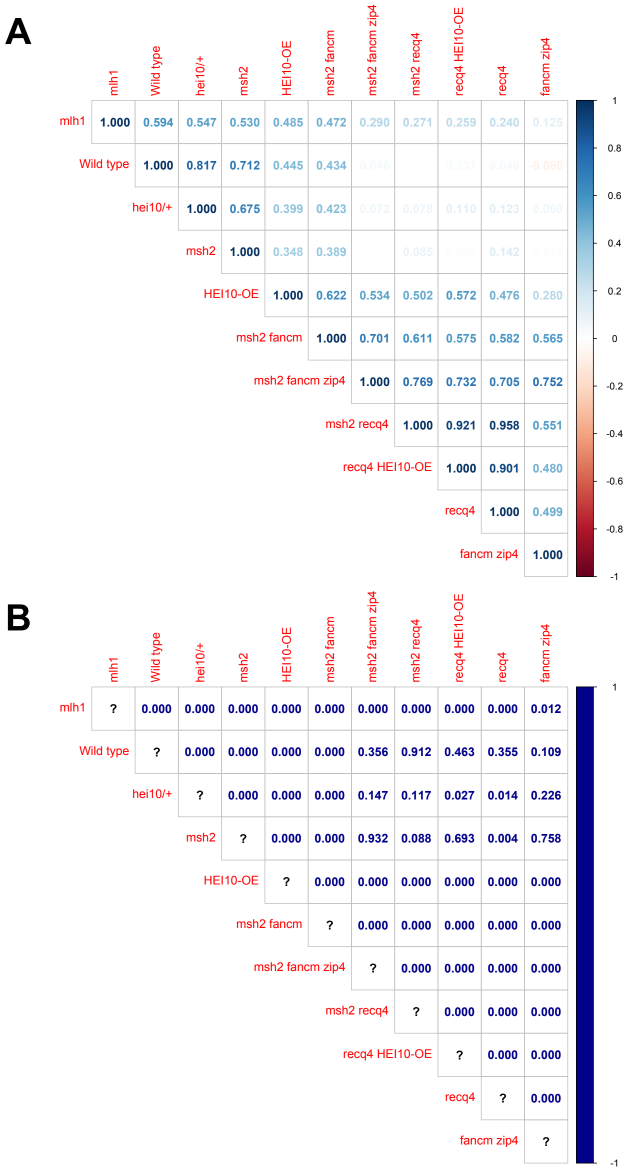

Supplementary Figure S6. Genome-wide correlation coefficient matrices of crossover distributions, calculated in adjacent 300 kb windows. Data for wild type, *msh2*, *HEI10-OE*, *msh2 fancm zip4*, *msh2 recq4*, *recq4 HEI10-OE*, *recq4*, and *fancm zip4* from refs. (17, 59, 63, 75, 76). **A.** Correlation coefficient values (Spearman's rank correlation). **B.** *P*-values.

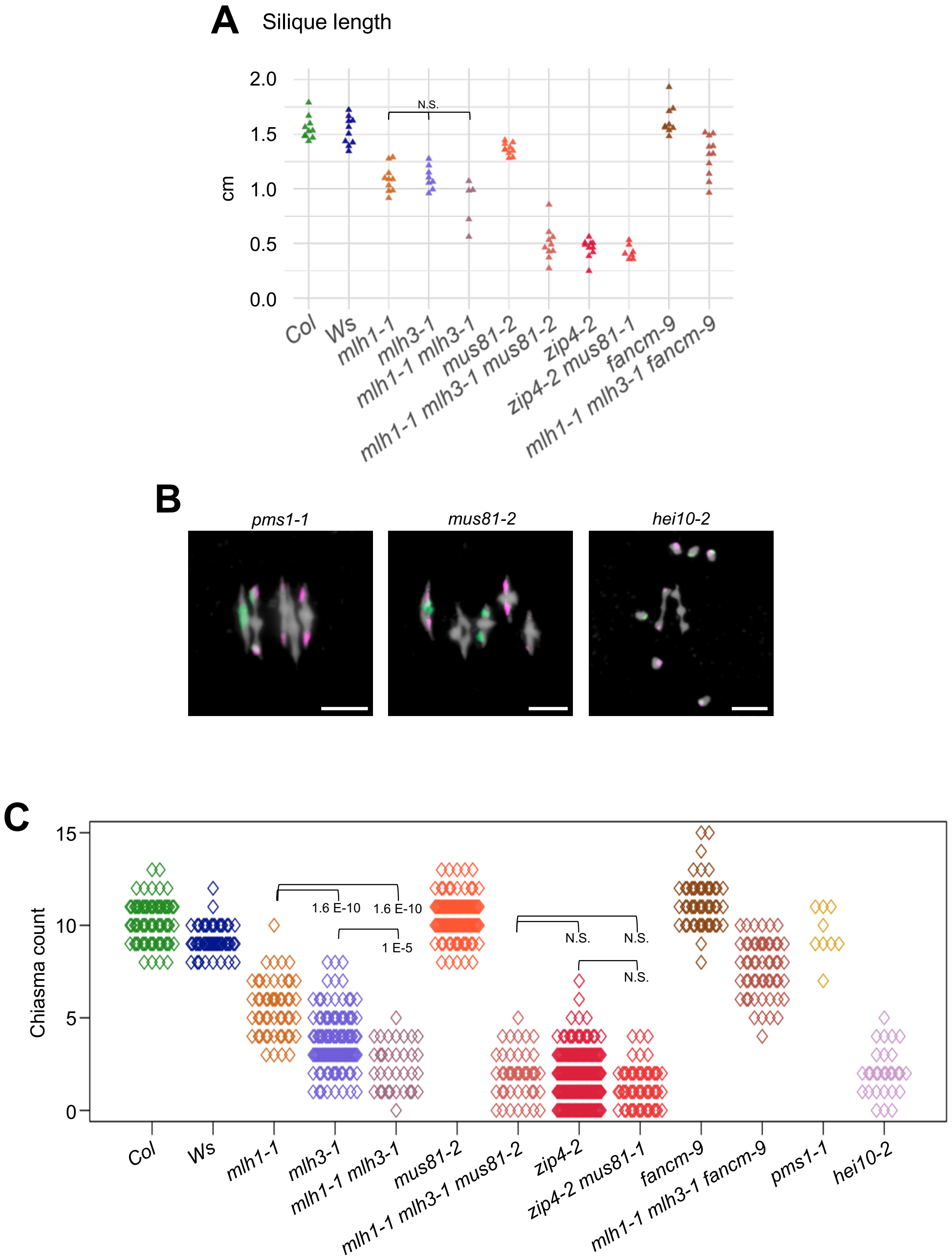

Supplementary Figure S7. Additional information for characterizing the genetic interaction between MutLγ loss of function and class II crossover recombination loss of function *mus81* and *fancm*. A. Fertility assessed by silique length. The phenotype is consistent with the seed set and pollen viability in Figure 6. The *P* values were estimated using one-way ANOVA and the Tukey HSD tests (Supplementary Tables S7-S8). n = 5 to 11. **B.**

Cytological characterization at metaphase I for *pms1, mus81*, and *hei10.* The *pms1* and *mus81* mutations do not affect bivalent formation, whereas *hei10,* as most *zmm* mutants, shows dramatically reduced chiasmata/ bivalents formation. **C.** Chiasmata count per meiocyte for all tested lines. The observed phenotype is consistent with the fertility assessment and cytological analysis. The *P* values were estimated using one-way ANOVA and the Tukey HSD/Tukey-Kramer tests (Supplementary Table S16). The number of characterized meiocytes are Col (n = 69), Ws (n = 50)*, mlh1* (n = 53)*, mlh3* (n = 108), *mlh1 mlh3* (n = 34), *mus81* (n = 102), *mlh1 mlh3 mus81* (n = 44), *zip4* (n=255), *zip4 mus81* (n=57), *fancm* (n = 52), *mlh1 mlh3 fancm* (n = 55), *pms1* (n= 9) and *hei10* (n= 29).

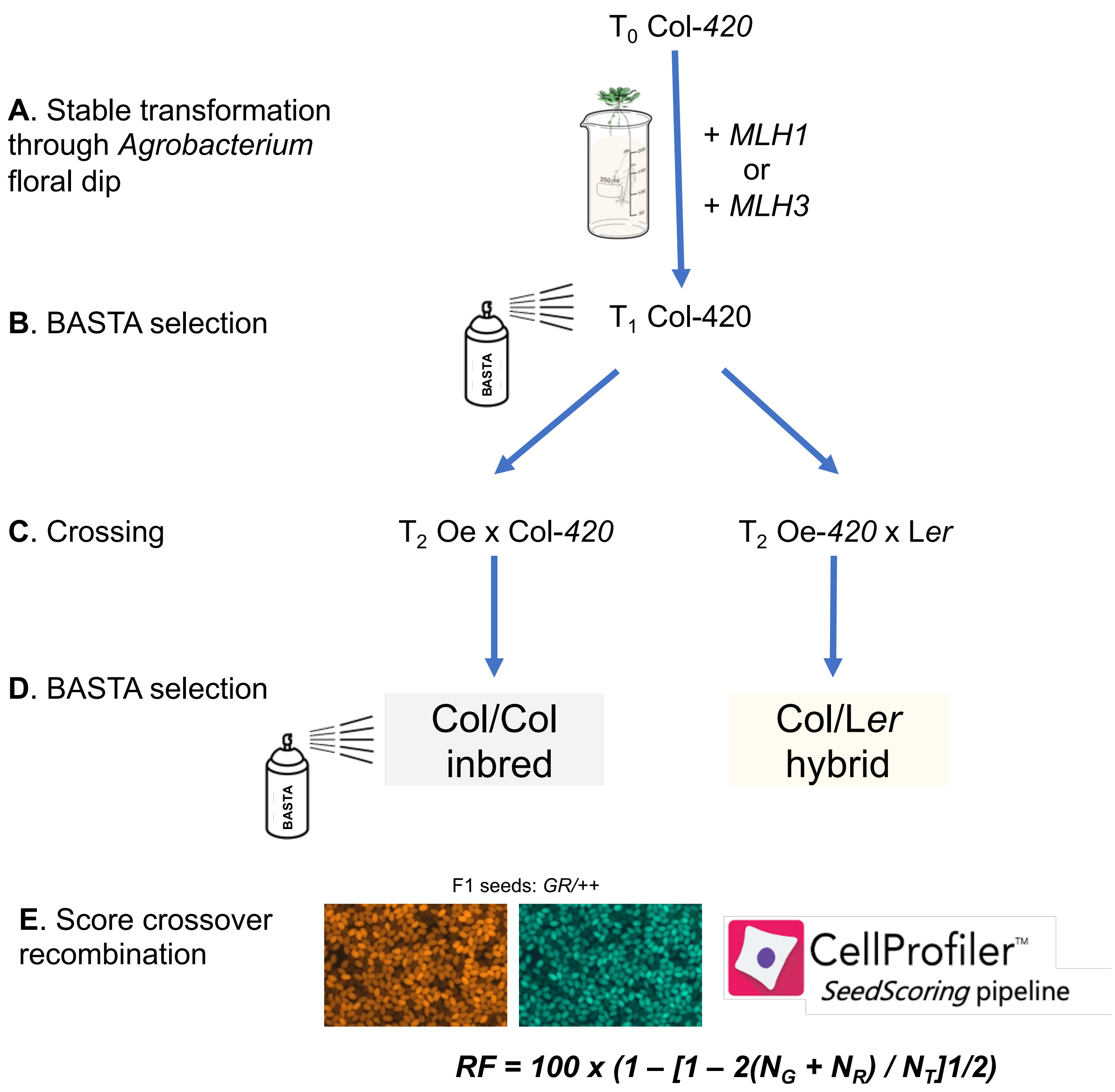

Supplementary Figure S8. Experimental design for measuring crossover frequency in Col/Col inbred and Col/*Ler* hybrid contexts, for MutL overexpressors. A. T_0 Col$^{420/+}$ plants were transformed with constructs harboring genomic sequences of *MLH1* or *MLH3* under their respective native promoters or the meiosis-specific DMC1 promoter. They were grown to seed and collected. **B.** T_1 seeds were sown and T_1 plants were selected for transformants using BASTA. T_1 transformants were grown to seed and collected. **C.** T_2 seeds were sown and T_2 plants were selected with BASTA then crossed to Col and L*er*, F1 seeds were collected. **D.** F_1 plants were grown, selected with BASTA, and collected. **E.** The obtained F_2 seeds were pictured following the protocol in (6) to measure crossover recombination frequency in the *420* interval.

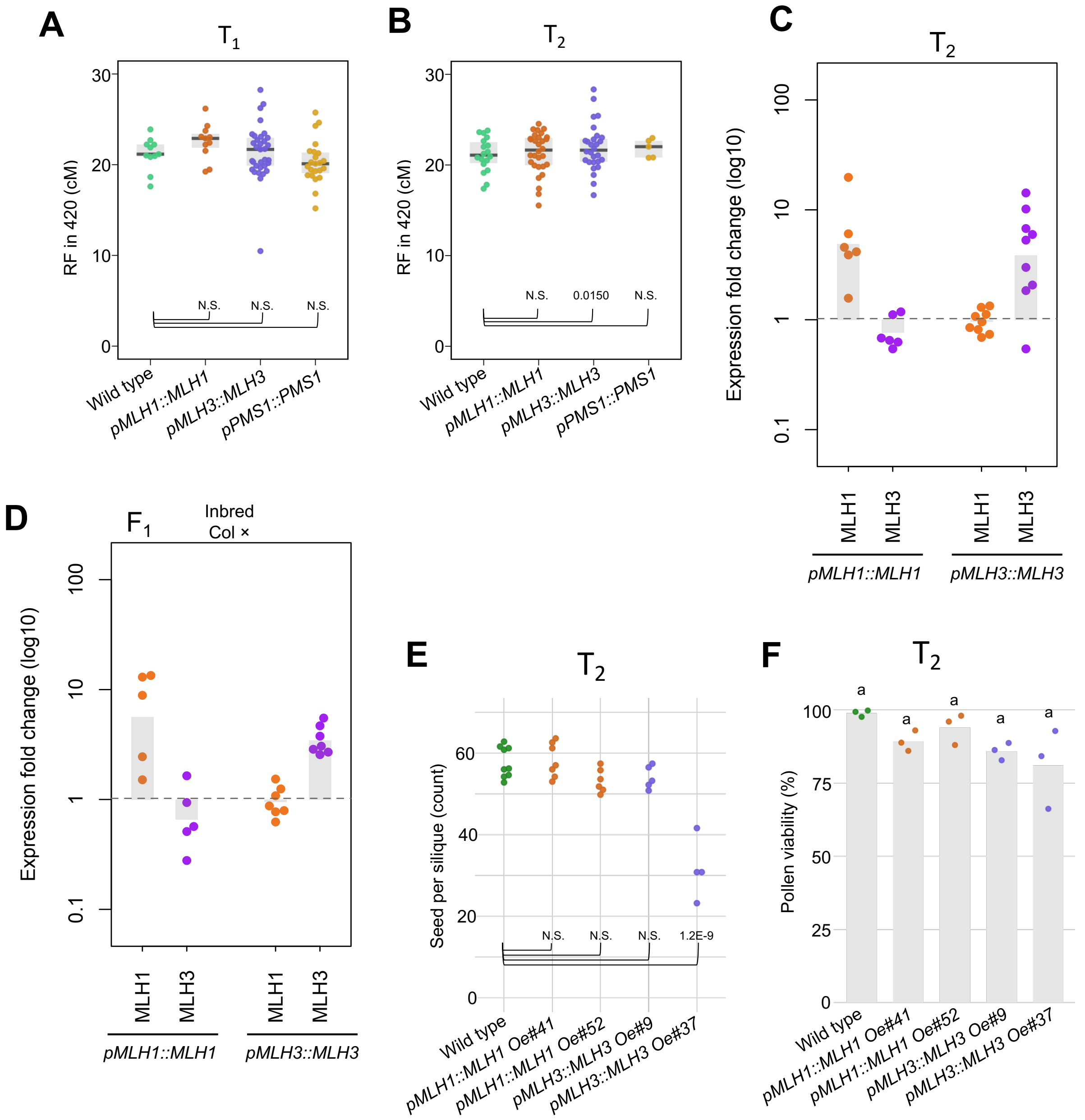

Supplementary Figure S9. Phenotypical characterization of *MutL* overexpressors under the control of their respective native promoters. A-B. Local meiotic crossover recombination frequency, measured in the *420* interval, for *MLH1, MLH3,* and *PMS1* in T_1 generation (**A**), and T_2 generation (**B**). Each dot represents a measurement from one individual. The center line of a boxplot indicates the median; the upper and lower bounds indicate the 75th and 25th percentiles, respectively, and the *P* values were estimated using the Welch t-test. **C.** Expression levels in T_2 plants carrying *pMLH1::MLH1* or *pMLH3::MLH3* constructs as determined via RT-qPCR for both *MLH1* and *MLH3* in each line. Each dot represents one biological replicate (one T_2 plant). Bar plots show the average fold change relative to wild type. **D.** As in C, but for F_1 progeny resulting from the cross of T_2 plants with wild-type Col. **E-F.** Fertility assays for *MLH1* and *MLH3* overexpressors were assessed by

seed set (**E**), and pollen viability (**F**). The *P* values were estimated using one-way ANOVA and the Tukey HSD tests (Supplementary Tables S19-S20). n = 4 to 9 in D and n = 3 in E. Expression level quantification using RT-qPCR and fertility assays were performed on T_2 generation plants.

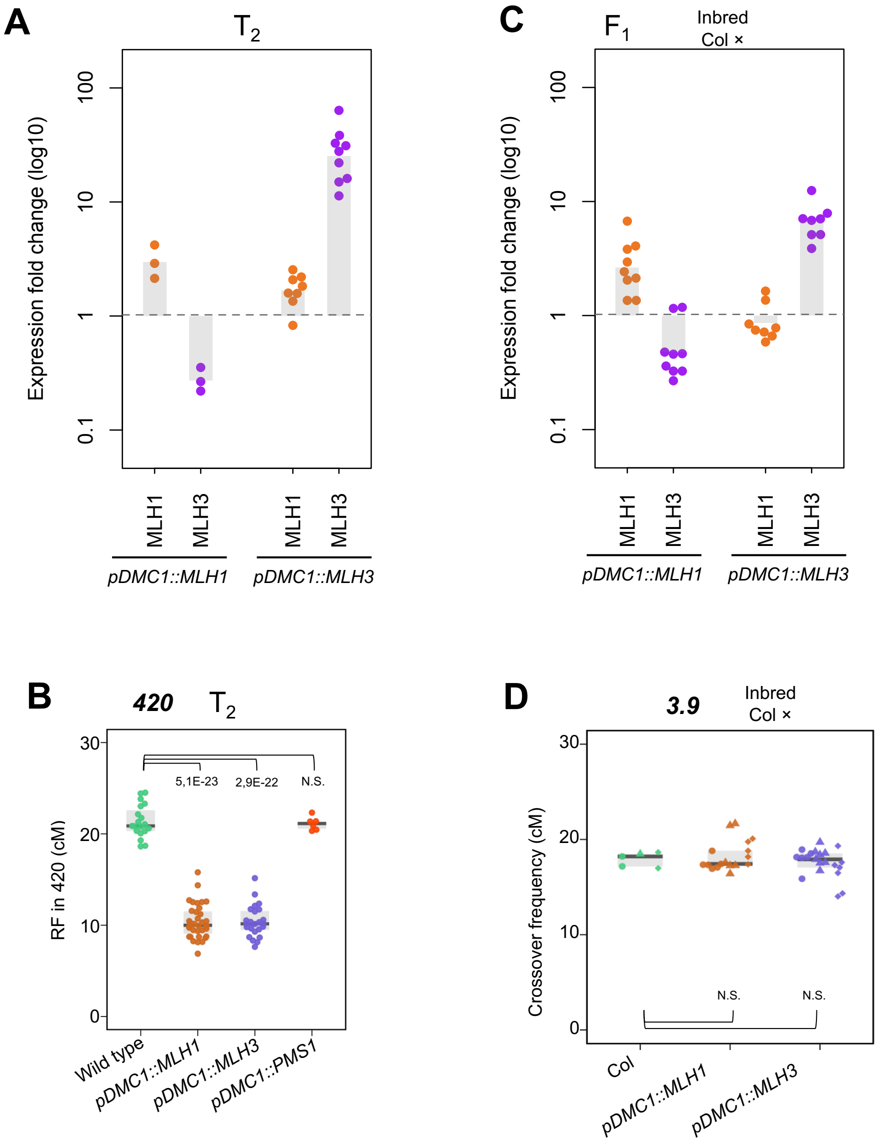

Supplementary Figure S10. Additional information on the phenotypical characterization of *MutL* overexpressors under the control of *DMC1* promoter. A. Expression levels in T_2 plants carrying *pDMC1::MLH1* or *pDMC1::MLH3* constructs as determined via RT-qPCR for both *MLH1* and *MLH3* for each line. Each dot represents one biological replicate (one T_2 plant). Bar plots show the average fold change relative to wild type. **B.** Local meiotic crossover recombination frequency, measured in the *420* interval, for *MLH1, MLH3,* and *PMS1* under *DMC1* promoter in T_2 generation. Each dot represents a measurement from one individual. The center line of a boxplot indicates the median; the upper and lower bounds indicate the 75th and 25th percentiles, respectively, and the *P* values were estimated using the Welch t-

test. **C.** As in (**A**), but for F_1 progeny resulting from the cross of T_2 plants with wild-type Col. **D.** Crossover frequency in inbred Col/Col and hybrid Col/L*er* F_1 plants overexpressing additional *MLH1* or *MLH3* copies under *DMC1* promoter, measured in the *420* interval and in Col/Col inbred plants in the *3.9* interval.

Supplementary Tables (in Excel file)

Supplementary Table S1. Genotyping primers used for discriminating the different used mutants.

Supplementary Table S2. CRISPR Cas9 gRNA and cloning primer sequences used for knocking out *MLH1.*

Supplementary Table S3. Primer sequences used for cloning MLH1, MLH3 and PMS1 under their respective native promoters or in-frame with the *DMC1* promoter for ectopic expression.

Supplementary Table S4. Summary of the sequencing data for Col/L*er mlh1* and *hei10-2/+.*

Supplementary Table S5. The qPCR primer sequences used to quantify the different specified targets following the reverse transcription of total RNA.

Supplementary Table S6. Summary table of the average numbers of univalents, bivalents, and chiasmata in the tested lines.

Supplementary Table S7. The average number of chiasmata per chromosome and Pollen Mother Cells (PMC) of MutL mutants in combination with class II factors at Metaphase I.

Supplementary Table S8. Meiotic behavior established from Pollen Mother Cells of MutL mutants in combination with class II factors at Metaphase I.

Supplementary Table S9. Chromosome configuration proportions in all tested lines. Submetacentric (1+3+5) and acrocentric (2+4) chromosomes behavior is discriminated.

Supplementary Table S10. Multidirectional One way ANOVA Tukey HSD test on seed set data in Figure 1b.

Supplementary Table S11. Multidirectional One way ANOVA Tukey HSD test on silique length data in Figure 1c.

Supplementary Table S12. Multidirectional One way ANOVA Tukey HSD test on pollen viability in Figure 1d.

Supplementary Table S13. Multidirectional One way ANOVA Tukey HSD test on seed set data in Figure 6a.

Supplementary Table S14. Multidirectional One way ANOVA Tukey HSD test on pollen viability data in Figure 6b.

Supplementary Table S15, Multidirectional One way ANOVA Tukey HSD test on mean chiasmata count data in Figure 6f.

Supplementary Table S16. Multidirectional One way ANOVA Tukey HSD test on silique length data in Supplementary Figure 6a.

Supplementary Table S17. Multidirectional One way ANOVA Tukey HSD test on chiasmata count data in Supplementary Figure 6c.

Supplementary Table S18. Multidirectional One way ANOVA Tukey HSD test on seed set data in Figure 7d.

Supplementary Table S19. Multidirectional One way ANOVA Tukey HSD test on pollen viability data in Figure 7e.

Supplementary Table S20. Multidirectional One way ANOVA Tukey HSD test of seed set data in Supplementary Figure 9d.

Supplementary Table S21. Multidirectional One way ANOVA Tukey HSD test on pollen viability data in Supplementary Figure 9e.
